# Geographic and Age Variations in Low Body Mass Index Among Community-Dwelling Older People in Xinjiang: A Cross-Sectional Study

**DOI:** 10.3389/fmed.2021.675931

**Published:** 2021-07-15

**Authors:** Jinling Liu, Qun Qu, Saiyare Xuekelati, Xue Bai, Li Wang, Hong Xiang, Hongmei Wang

**Affiliations:** ^1^Second Department of the Cadre Health Care Center, People's Hospital of Xinjiang Uygur Autonomous Region, Urumqi, China; ^2^The Health Center for the Cadre of Xinjiang Uygur Autonomous Region, Urumqi, China

**Keywords:** undernutrition, BMI, aging, community-dwelling older people, Xinjiang

## Abstract

**Background:** Studies have shown an association between undernutrition and increased adverse outcome, as well as substantial geographic and age variations in undernutrition. Body mass index (BMI), a core indicator of undernutrition, is easy to measure and reflects the nutritional and health status of the human body. It is a simple and suitable tool for epidemiological investigations in large sample populations. Herein, we provide the first description of geographic and age variations in the prevalence of low BMI among community-dwelling older people in Xinjiang.

**Methods:** From January 2019 to December 2019, using a multi-stage random sampling method, we conducted a cross-sectional epidemiological survey of the community-dwelling older people in Xinjiang at different latitudes. Of the 87,000 participants, the statistical analyses included 86,514 participants with complete data.

**Results:** In Xinjiang, the prevalence of low BMI was 7.7% in the community-dwelling older people. The BMI gradually decreased with increasing age and gradually increased with latitude. The prevalence of low BMI in northern Xinjiang was 5.3%, which was significantly lower than that in eastern (7.7%) and southern (9.3%) Xinjiang. In the 60–69-, 70–79-, 80–89-, and ≥90-year age groups, the prevalence rates of low BMI were 5.8, 7.9, 10.0, and 13.9%, respectively. After adjusting for confounding factors (sex, ethnic group, hypertension, diabetes, hyperlipemia, smoking, and drinking), multivariate logistic regression analysis showed that the odds ratios (95% CI) for low BMI in eastern and southern Xinjiang were 1.165 (1.056–1.285) and 1.400 (1.274–1.538), respectively, compared to northern Xinjiang. The adjusted odds ratios (95% CI) for low BMI in the 70–79-, 80–89-, and ≥90-year age groups were 1.511 (1.39–1.635), 2.233 (2.030–2.456), and 3.003 (2.439–3.696), respectively, compared to the 60–69-year age group.

**Conclusion:** The results of this study revealed geographic and age variations in the prevalence of low BMI in the community-dwelling older people in Xinjiang. The prevalence of low BMI gradually increased as the latitude decreased and as age increased.

## Introduction

Research on aging has attracted significant attention worldwide. The World Health Organization has reported a rapidly aging global population. In the first 50 years of the 21st century, the proportion of the older population (≥60 years of age) globally is expected to increase from 11 to 22%, corresponding to an increase from 605 million to two billion individuals (1). In China, by the end of 2018, 17.9% of the population was aged ≥60 years; 11.9% of whom were aged ≥65 years. Hence, China is gradually becoming a super-aged society (2).

With aging, older individuals experience a series of inevitable nutritional problems such as chewing and swallowing difficulties caused by tooth loss, absorption and utilization obstacles due to decreased body tissue and organ function, anorexia caused by medication, and other diseases. As a result, the energy and nutrient intake of older people cannot meet the needs of their bodies, eventually leading to undernutrition or a risk of undernutrition. The “Nutrition and Health Report of the Chinese Older People (2015)” revealed the high nutritional risk of older individuals in China, with up to 48.4% of individuals having a poor nutritional status (2, 3). In 2012, the total economic burden associated with undernutrition among older people in China was 84.14 billion Yuan, of which the direct economic burden was 63.93 billion Yuan. This accounted for 10.6% of the treatment cost of all diseases among older people, thereby increasing the financial burden of millions of families, as well as other countries (4, 5). Therefore, undernutrition in older individuals requires attention.

Numerous screening tools have been developed to assess the presence and risk of undernutrition. Body mass index (BMI), a common indicator, is easy to measure and reflects the nutritional and health status of the human body. It is simple and suitable for use in epidemiological investigations in large sample populations. Among the numerous consensus or guidelines published at present, a low BMI is a relatively consistent standard measurement (or diagnostic criterion), which has always been the core index of undernutrition assessment (or diagnosis).

Previous studies have confirmed that compared to older people with good nutritional status, those with undernutrition or high undernutrition risk show low immunity, poor resistance, decreased quality of life, prolonged hospital stay, increased hospital costs, poor treatment effect, poor prognosis, and high incidence of complications (6–9). Numerous studies have demonstrated the association of undernutrition with a high incidence of and mortality due to fractures and various diseases in older individuals (10–15). Therefore, considering the increasing proportion of older individuals in China, the “Healthy China 2030 Program Outline” and improvements in the nutritional status and health of all citizens in China need to be implemented.

The prevalence of undernutrition can vary substantially based on geography and age. Thus, we hypothesized that the prevalence of undernutrition in older individuals differs in Xinjiang province due to geographic factors. Identifying variations in undernutrition among regions can help direct prevention efforts. Province-level maps are often created based on data available in each province. However, maps of undernutrition prevalence cannot identify disparities within a province. Although many epidemiological studies on undernutrition have been conducted in recent years, geographic and age variations in undernutrition prevalence in the older population in Xinjiang are unknown. Therefore, epidemiological surveys of the older population in Xinjiang are needed.

This cross-sectional, epidemiological investigation was conducted among the community-dwelling older people in Xinjiang to describe the geographic and age differences in the prevalence of low BMI, the core index of undernutrition assessment (or diagnosis), in this population. Thus, this study may provide a reference for the formulation of prevention and treatment strategies for undernutrition in the community-dwelling older people in Xinjiang.

## Materials and Methods

### Study Participants

This cross-sectional study of the community-dwelling older people in Xinjiang was reviewed and approved by the Ethics Committee of People's Hospital of Xinjiang Uygur Autonomous Region. All research participants provided written informed consent.

**Figure 1:**
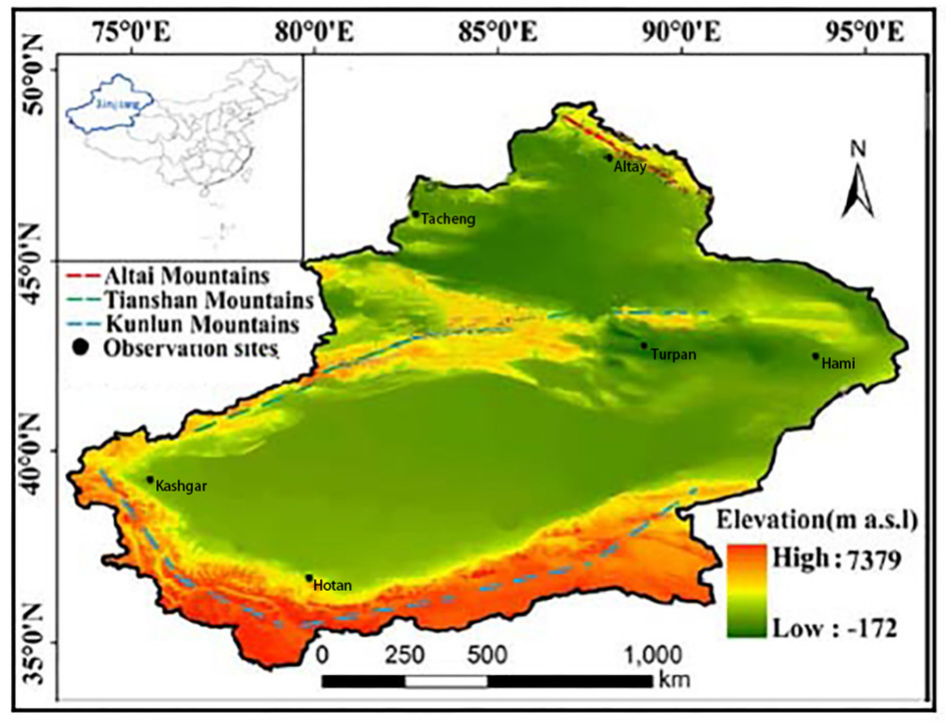
Observation sites in the study, southern Xinjiang, northern Xinjiang, and eastern Xinjiang, China.

Located in the hinterland of the Eurasia continent (73°40′–96°23′ E and 34°25′–48°10′ N), Xinjiang is divided into three distinct subregions (north, south, and east) according to its natural settings and climate patterns (16). From January to December 2019, a multilevel random sampling method was used to perform surveys of older individuals in Xinjiang. During the first stage, two regions were randomly selected from the northern (Altai and Tacheng, located at latitudes of 46°21′−48°00′ N), eastern (Hami and Turpan, located at latitudes of 40°52′−41°12′ N), and southern (Hotan and Kashgar, located at latitudes of 35°28′−37°12′ N) Xinjiang subregions (Figure 1). During the second stage, one city was randomly selected from each region. During the third stage, two counties were randomly selected from each city. During the fourth stage, 7,500 older individuals aged 60 years or older were randomly selected from each county. A total of 90,000 study participants were selected.

**Figure 2:**
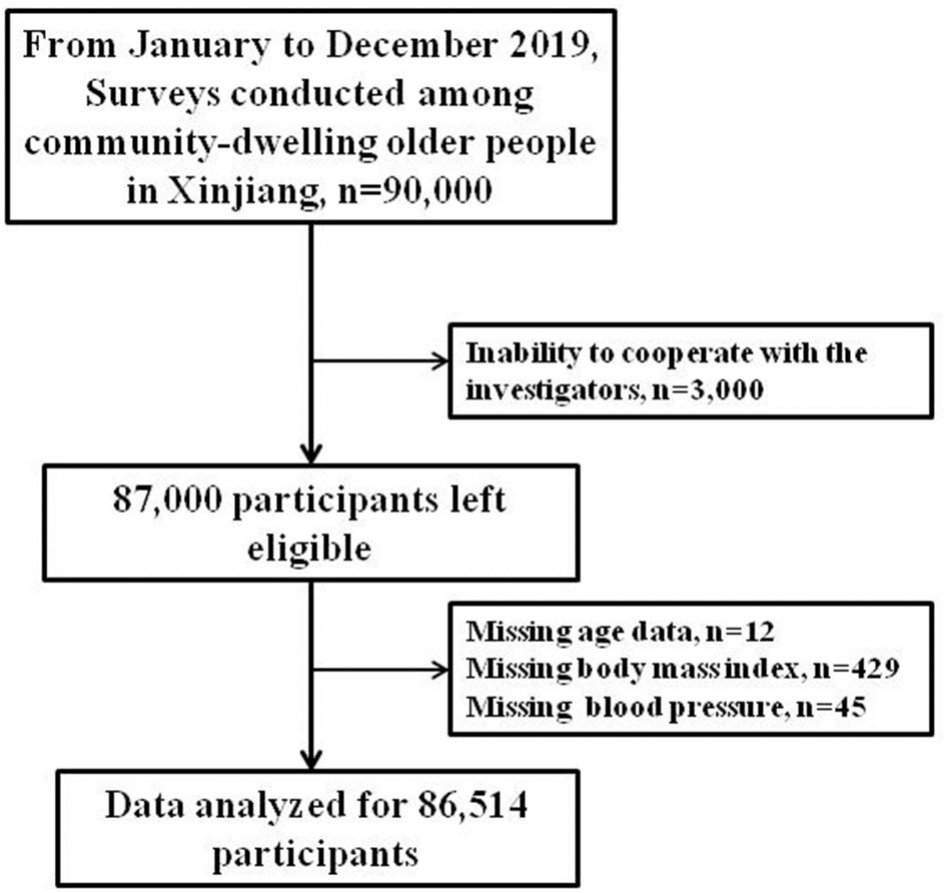
Selection procedure of the study participants.

The inclusion criteria were age 60 years or older and the ability to complete the survey, understand the investigation, cooperate with the investigators, and provide signed informed consent. The exclusion criterion was the inability to cooperate with the investigators. A total of 87,000 individuals completed the survey, resulting in a response rate of 96.67%. We excluded 486 participants with incomplete data; therefore, the statistical analyses included data from a total of 86,514 participants (Figure 2).

### Questionnaire Survey

A set of standardized questionnaires was completed under the physicians' guidance. The questionnaire survey included information on age, sex, ethnic group, education level, occupation, disease history, medication history, family history, drinking history, smoking history, and eating habits.

### Physical Examination

Each participant underwent a detailed physical examination performed by a physician. Indicators such as blood pressure, weight, and height, were measured by qualified professionals using standard methods.

#### Blood Pressure Measurement

Systolic blood pressure (SBP) and diastolic blood pressure (DBP) were measured according to the methods recommended by the American Heart Federation. All participants were prohibited from smoking and drinking alcohol, tea, and coffee 30 min before blood pressure measurement. A desktop mercury sphygmomanometer was used to measure blood pressure. The blood pressure of the right upper arm of the participants in a seated position was measured by a medical specialist after a 10-min rest period. The blood pressure was measured three times consecutively every 2 min, at a room temperature of >18°C.

#### Weight Measurement

The participants were required to not eat before undergoing measurements, to remove their shoes, and to wear light clothing. A calibrated medical scale was used for weight measurement. The participants were asked to relax and to stand upright in the middle of the chassis of the scale, which had a reading accuracy of 0.1 kg.

#### Height Measurement

A measuring ruler, with a minimum scale of 1 mm, was fixed vertically to the ground. The participants were instructed to stand upright with both heels together and with the shoulders and hips close to the measuring ruler. The surveyor placed a square ruler on top of the participants' head, with the side of the right angle near the measuring ruler and the other side near the participant's scalp. The measuring ruler was accurate to 1 mm. BMI was calculated as follows: BMI = weight (kg)/height (m) ^2^.

### Collection of Blood Samples and Laboratory Testing

Disposable blood collection equipment was used to draw 10 mL of peripheral venous blood in the morning from the participants who had fasted for at least 10 h. Plasma (serum) was immediately centrifuged and separated from the blood cells and stored at −80°C for testing. Levels of fasting blood glucose (FBG), total cholesterol (TC), triglycerides (TG), high-density lipoprotein cholesterol (HDL-c), low-density lipoprotein cholesterol (LDL-c), serum creatinine (SCr), and serum urea nitrogen (SUN), and other biochemical indicators were examined within 1 month at the Clinical Laboratory Center of People's Hospital of Xinjiang Uygur Autonomous Region (a third-grade class A hospital) using a Hitachi 7600 automatic biochemical analyzer.

### Related Diagnostic Criteria

#### Low BMI

According to the undernutrition assessment (diagnosis) criteria for Asians from the “Global Leadership Initiative on Malnutrition Consensus (2018),” participants aged <70 years with a BMI of <18.5 kg/m^2^ or those aged ≥70 years with a BMI of <20 kg/ m^2^ were considered to have a low BMI.

#### Overweight/Obesity

According to the standard Chinese guidelines for adults for the prevention and control of overweight and obesity, a BMI of 24.0 kg/m^2^ ≤ BMI <28.0 kg/m^2^ was used to indicate “overweight” and a BMI ≥28.0 kg/m^2^ was used to indicate “obesity.”

#### Hypertension

Hypertension was defined as an SBP of ≥140 mmHg and/or a DBP of ≥90 mmHg or a previous hypertension diagnosis/treatment for hypertension.

#### Diabetes Mellitus

Diabetes mellitus was defined as an FBG level of ≥7.0 mmol/L or a previous diagnosis of type 2 diabetes mellitus requiring treatment.

#### Hyperlipidemia

According to the 2016 Prevention and Treatment Guidelines for Chinese Adults with Dyslipidemia, hyperlipidemia was defined as a TC level of >5.18 mmol/L, an LDL-C level of >3.37 mmol/L, or a TG level of >1.7 mmol/L.

#### Smoking and Drinking

According to the standards proposed by the World Health Organization, smokers were defined as participants who smoked continuously or had smoked for a cumulative period of at least 6 months, while drinkers were defined as those who drank alcohol once weekly, with an alcohol consumption of 8 g per week.

### Quality Control

Standard methods and unified and standardized instruments (checked and corrected by professionals) were used to collect the relevant data. To control for observer error, all investigators underwent training on conducting accurate questionnaire surveys (the questionnaire survey was performed by a medical specialist). Furthermore, physical examinations; blood sample collection, transportation, separation, preservation, and marking; questionnaire review, data entry, etc. were all completed by specialists. Only qualified surveyors participated in the survey. According to the principle of double-blind data entry, two professionals entered the data in parallel and performed the statistical analyses after verification.

### Statistical Analysis

Data analyses were performed using IBM SPSS Statistics for Windows, version 19.0 (IBM Corp., Armonk, NY, USA). Values were expressed as mean ± standard deviation. Counting data were expressed as percentages. The distributions of participants' characteristics between the low BMI, normal BMI, and overweight and obesity groups were analyzed using one-way analysis of variance (ANOVA) or chi-square tests. The differences in BMI and the prevalence of low BMI among individuals of different ages and geographic regions were also analyzed by one-way ANOVA or chi-square tests. After adjusting for confounding factors (age, sex, ethnic group, hypertension, diabetes, hyperlipemia, smoking, drinking), multivariate logistic regression was used to analyze the influence of geographic variations on low BMI. *P* < 0.05 was considered statistically significant.

## Results

### Clinical Characteristics

Table 1 presents the clinical characteristics of the study participants. Age, SBP, DBP, TC, TG, LDL-c, HDL-c, alanine aminotransferase, SCr, SUN, FBG, sex, smoking, drinking, hypertension, diabetes, hyperlipemia, geographic region, and ethnic group differed significantly between the low BMI, normal BMI, and overweight and obesity groups (all *P* < 0.001). No significant differences in aspartate aminotransferase levels were observed between the three groups (*P* = 0.221).

**Table 1:** Clinical characteristics of the study participants.

	**Normal BMI**	**Low BMI**	**Overweight/obesity**	***F(*χ*2)***	***P***
	**group (*n* = 24,997)**	**group (*n* = 6,662)**	**group (*n* = 54,855)**		
Age (year)	74.01 ± 6.309	74.76 ± 6.548	73.04 ± 5.767	397.163	<0.001
SBP (mmHg)	131.9 ± 20.047	127.34 ± 20.754	137.98 ± 20.009	1361.266	<0.001
DBP (mmHG)	75.20 ± 12.068	73.27 ± 12.242	78.37 ± 12.228	924.282	<0.001
TC (mmol/L)	4.65 ± 1.300	4.49 ± 1.280	4.81 ± 1.304	275.672	<0.001
TG (mmol/L)	1.31 ± 0.856	1.15 ± 0.866	1.60 ± 1.031	1141.382	<0.001
HDL-c (mmol/L)	1.44 ± 0.809	1.48 ± 0.562	1.38 ± 0.854	57.806	<0.001
LDL-c (mmol/L)	2.57 ± 1.007	2.44 ± 0.923	2.68 ± 0.979	174.621	<0.001
ALT (U//L)	19.79 ± 13.166	18.37 ± 12.785	22.14 ± 13.746	137.297	<0.001
AST (U//L)	23.02 ± 10.737	23.60 ± 10.984	22.80 ± 10.338	1.509	0.221
SCr (umol/L)	73.36 ± 25.033	71.96 ± 22.871	74.07 ± 24.663	24.26	<0.001
SUN (mmol/L)	5.74 ± 2.534	5.92 ± 3.113	5.63 ± 2.465	46.301	<0.001
FBG (mmol/L)	5.66 ± 1.855	5.40 ± 1.590	6.16 ± 2.248	742.835	<0.001
Sex (Male, %)	51.8	47.2	45.8	246.889	<0.001
Smoking (Yes, %)	10.3	9.4	9.0	33.023	<0.001
Drinking (Yes, %)	5.0	3.7	5.8	60.349	<0.001
Hypertension (Yes, %)	43.0	34.3	57.5	2307.624	<0.001
Diabetes (Yes, %)	11.5	7.3	20.6	1447.149	<0.001
Hyperlipemia (Yes, %)	43.2	34.9	54.5	1073.696	<0.001
**Geographic region**					
Northern Xinjiang (%)	25.5	20.2	31.8	663.366	<0.001
Eastern Xinjiang (%)	28.3	27.3	26.7		
Southern Xinjiang (%)	46.2	52.7	41.5		
**Ethnic group**					
Han (%)	58.8	67.6	52.9	850.339	<0.001
Uygur (%)	32.3	24.5	33.8		
Kazakh (%)	4.1	3.7	7.0		
Others (%)	4.8	4.2	6.3		

*BMI, body mass index; SBP, systolic blood pressure; DBP, diastolic blood pressure; TC, total cholesterol; TG, triglycerides; HDL-c, high density lipoprotein-cholesterol; LDL-c, low density lipoprotein-cholesterol; ALT, alanine aminotransferase; AST, aspartate aminotransferase; SCr, serum creatinine; SUN, serum urea nitrogen; FBG, fasting blood glucose*.

### Geographic and Age Variations in the Prevalence of Low BMI Among the Community-Dwelling Older People in Xinjiang

Overall, the prevalence of low BMI was 7.7% in community-dwelling older people of Xinjiang. Table 2 shows the geographic and age disparities of BMI and the prevalence of low BMI in the population. The BMI gradually decreased as age increased and gradually increased with latitude. The prevalence of low BMI in northern Xinjiang was 5.3%, which was significantly lower than that in eastern (7.7%) and southern (9.3%) Xinjiang. In the 60–69-, 70–79-, 80–89-, and ≥90-year age groups, the prevalence rates of low BMI were 5.8, 7.9, 10.0, and 13.9%, respectively.

**Table 2:** Geographic and age disparities of low BMI in the community-dwelling older people in Xinjiang.

	**BMI (kg/m^**2**^)**	**Prevalence of low BMI (%)**
**Geographic region**		
Counties in northern Xinjiang (46°21′−48°00′ N)	26.29 ± 4.310	5.3
Counties in eastern Xinjiang (40°52′−41°12′ N)	25.57 ± 4.568	7.7
Counties in southern Xinjiang (35°28′−37°12′ N)	25.32 ± 4.011	9.3
*F (χ2)*	332.490	326.46
*P*	<0.001	<0.001
**Age group (year)**		
60–69	26.22 ± 4.405	5.8
70–79	25.69 ± 4.366	7.9
80–89	24.92 ± 4.145	10
≥90	24.05 ± 4.018	13.9
*F (χ2)*	326.987	306.322
*P*	<0.001	<0.001

After adjusting for confounding factors (sex, ethnic group, hypertension, diabetes, hyperlipemia, smoking, and drinking), multivariate logistic regression analysis showed that the odds ratios [95% confidence intervals (CIs)] for low BMI in eastern (40°52′−41°12′ N) and southern (35°28′−37°12′ N) Xinjiang were 1.165 (1.056–1.285) and 1.400 (1.274–1.538), respectively, compared to counties in northern Xinjiang (46°21′−48°00′ N) (Table 3). The adjusted odds ratios (95% CI) for low BMI in the 70–79-, 80–89-, and ≥90-year age groups were 1.511 (1.39–1.635), 2.233 (2.030–2.456), and 3.003 (2.439–3.696), respectively, compared to the 60–69-year age group (Table 3).

**Table 3:** Adjusted odds ratios (95% confidence intervals) of low BMI vs. non-low BMI associated with age and latitude.

	**B**	**Standard error**	**Odds ratios**	**95% Confidence intervals**	***P***
Age group (year)^*^					
60–69			1.000		<0.001
70–79	0.413	0.040	1.511	1.397–1.635	<0.001
80–89	0.803	0.049	2.233	2.030–2.456	<0.001
≥90	1.099	0.106	3.003	2.439–3.696	<0.001
Latitude group^**^					
46°21′−48°00′ N			1.000		<0.001
40°52′−41°12′ N	0.153	0.050	1.165	1.056–1.285	0.002
35°28′−37°12′ N	0.336	0.048	1.400	1.274–1.538	<0.001

* *The odds ratios for age grouping are adjusted for latitude, sex, ethnic group, hypertension, diabetes, hyperlipemia, smoking and drinking status*.

** *The odds ratios for latitude grouping are adjusted for age, sex, ethnic group, hypertension, diabetes, hyperlipemia, smoking and drinking status*.

*BMI, body mass index*.

## Discussion

The results of the present study revealed the varying prevalence of low BMI among the community-dwelling older people across Xinjiang. The prevalence of low BMI gradually increased with decreasing latitude (5.3% in northern Xinjiang, 7.7% in eastern Xinjiang, and 9.3% in southern Xinjiang). Moreover, the prevalence of low BMI increased gradually with age (5.8, 7.9, 10.0, and 13.9% in the 60–69-, 70–79-, 80–89-, and ≥90-year age groups, respectively). The results reveal the geographic and age disparities in low BMI among the community-dwelling older people in Xinjiang.

### Age Differences in Low BMI

In the older population, as age increases, food intake decreases; this is affected by factors such as society, psychology, physiology, drugs, and behavior, and ultimately leads to a high incidence of undernutrition (17, 18). The reported risk of undernutrition in the people aged ≥60 years in China was 5.4%, while that in the hospitalized older individuals has been increasing continuously up to 50–70% (17, 19). Studies also revealed the presence of undernutrition in 5–30% of older people living at home, 6–70% of older people in nursing homes, and 20–60% of older inpatients (20, 21). The results of previous studies also confirmed the positive correlation between undernutrition prevalence and age; thus, undernutrition is also considered a geriatric syndrome (22, 23). The present study used a multi-stage random sampling method to perform an epidemiological survey of low BMIin the community-dwelling older people in Xinjiang. The prevalence of low BMI in this population was 7.7% and the prevalence of low BMI increased with age, indicating the need to screen for undernutrition and provide reasonable nutritional interventions in the older people.

### Geographic Variations of Low BMI

Disease is not distributed randomly in the population; rather, it shows time and space distributions. Spatial attributes provide data related to the spatial entity and geographical location. The incidence and prevalence of disease in certain populations, climates, environments, are all related to the geographical location. Geographic variations in undernutrition and BMI have been documented in children, adolescents, adults, and older residents in nursing homes (24–27).

According to the “China Obesity Index Survey (2015),” the distribution of obesity (BMI ≥28.0 kg/m^2^) in China decreased from north (high latitude) to south (low latitude). The prevalence of obesity in northern China (35%) was higher than that in southern China (27%). The main reasons for the higher BMI include higher latitude and colder weather, as well as individual factors such as less exercise, slower metabolism, and fat deposition. In contrast, lower latitude and hotter weather, as well as individual factors including increased exercise and faster metabolism were associated with a lower BMI. Moreover, the prevalence of obesity (increased BMI) gradually decreased in the Chinese urban population with decreasing latitude (28). Similarly, in this study, the prevalence of low BMI increased gradually as the latitude decreased (southern Xinjiang (35°28′−37°12′ N)> eastern Xinjiang (40°52′−41°12′ N)> northern Xinjiang (46°21′−48°00′ N).

The geographic variation may be related to objective factors such as regional climate and lifestyle of local residents at different latitudes.

The survey sites in northern Xinjiang (46°21′−48°00′ N) experience only cold and warm seasons a year. Winter lasts for 6 months, with the coldest temperature of −40°C. Summer lasts only briefly, with the potential for snowfall even during this season and a frost-free period of <90 days per year. The summer season is characterized by rain, humidity, and cool temperatures. In contrast, the survey sites in eastern Xinjiang (40°52′−41°12′ N) are characterized by a continental arid desert climate with long periods of sunshine, high temperatures, large temperature fluctuations between day and night, little precipitation, and strong winds. The annual average temperature is 14°C, while that in summer is approximately 30°C. Annually, eastern Xinjiang has an average of 99 hot days with temperatures >35°C and 28 days with temperatures >40°C. The survey sites in southern Xinjiang (35°28′−37°12′ N) have an arid climate with extreme droughts within a warm temperate zone. Temperatures are high in the summer and moderate in the winter, with little precipitation, sufficient light, abundant heat, long frost-free periods, large temperature differences between day and night, and average annual precipitation of 35 mm (29).

Thus, such unique geographical environments result in unique lifestyle among the local residents. The residents of northern Xinjiang have higher alcohol, beef, mutton, and spicy food consumption and lower vegetable consumption. These participants also preferred high-fat and high-protein diets (such as meat and dairy food) (30). Because the weather is not suitable for sports, these individuals exercise less often, which results in fat accumulation and subsequent high BMI. In contrast, residents in southern Xinjiang mainly consume carbohydrates such as dried fruits and wheat flour (30). The relatively warm weather is conducive for frequent exercise; hence, fat is not easily accumulated, leading to a high incidence of low BMI. It was reported that the residents in the eastern Xinjiang have a higher level of consumption of vegetables and fruits, compared to those in other regions (30). Vegetables and fruits are rich and contain a higher proportion of sugar in this region, which are mainly benefited from the huge difference in temperature between day and night. Moreover, the high temperature in summer in this area leads to decreased exercise and consequently increases the risk of fat accumulation. Therefore, geographic variations in the prevalence of low BMI may be due to geographic variations in other lifestyle risk factors. The various potential causes of geographic variations require further investigation to determine the contribution of each of those causes.

Our study has some notable limitations. First, we did not collect data on the participants' diet and exercise, which may be related to low BMI. Second, additional nutritional assessments were not performed; hence, we were unable to obtain further information on the prevalence of undernutrition in this population.

Despite these limitations, our study has several strengths. This is the first epidemiological survey of low BMI in Xinjiang with a large sample size. Owing to the multi-stage random sampling strategy used in this study, we were able to obtain a representative sample population and uniform data coverage across continental Xinjiang.

We have provided evidence of geographic and age differences in the prevalence of low BMI among the community-dwelling older people in Xinjiang. Our results confirmed that the prevalence of low BMI gradually increased with decreasing latitude and with increasing age. This information can be used to establish prevention efforts targeted at particular populations within Xinjiang.

## Data Availability Statement

The original contributions presented in the study are included in the article/supplementary material, further inquiries can be directed to the corresponding author/s.

## Ethics Statement

The studies involving human participants were reviewed and approved by Ethics Committee of People's Hospital of Xinjiang Uygur Autonomous Region. The patients/participants provided their written informed consent to participate in this study.

## Author Contributions

HW contributed to the study design, data collection and analysis, result interpretation, and provided critical manuscript revisions. QQ and JL wrote the article. SX, XB, LW, and HX were involved in the data analysis and interpretation and also provided critical manuscript revisions. All authors read and approved the submitted manuscript.

## Conflict of Interest

The authors declare that the research was conducted in the absence of any commercial or financial relationships that could be construed as a potential conflict of interest.
